# Thermally-actuated microfluidic membrane valve for point-of-care applications

**DOI:** 10.1038/s41378-021-00260-3

**Published:** 2021-06-15

**Authors:** Muhsincan Sesen, Christopher J. Rowlands

**Affiliations:** Department of Bioengineering, Imperial College London, London, SW7 2AZ UK

**Keywords:** Engineering, Chemistry

## Abstract

Microfluidics has enabled low volume biochemistry reactions to be carried out at the point-of-care. A key component in microfluidics is the microfluidic valve. Microfluidic valves are not only useful for directing flow at intersections but also allow mixtures/dilutions to be tuned real-time and even provide peristaltic pumping capabilities. In the transition from chip-in-a-lab to lab-on-a-chip, it is essential to ensure that microfluidic valves are designed to require less peripheral equipment and that they are transportable. In this paper, a thermally-actuated microfluidic valve is presented. The valve itself is fabricated with off-the-shelf components without the need for sophisticated cleanroom techniques. It is shown that multiple valves can be controlled and operated via a power supply and an Arduino microcontroller; an important step towards transportable microfluidic devices capable of carrying out analytical assays at the point-of-care. It is been calculated that a single actuator costs less than $1, this highlights the potential of the presented valve for scaling out. The valve operation is demonstrated by adjusting the ratio of a water/dye mixture in a continuous flow microfluidic chip with Y-junction channel geometry. The power required to operate one microfluidic valve has been characterised both theoretically and experimentally. Cyclical operation of the valve has been demonstrated for 65 h with 585 actuations. The presented valve is capable of actuating rectangular microfluidic channels of 500 μm × 50 μm with an expected temperature increase of up to 5 °C. The fastest actuation times achieved were 2 s for valve closing (heating) and 9 s for valve opening (cooling).

## Introduction

Engineered valves lie at the heart of fluidics, especially microfluidics. Valves, just like transistors in electrical circuits, are one of the most important and commonly used tools for designing microfluidic platforms^1^ paving the way for programmability, automation and large-scale integration in microfluidics^2^.

Valves underpin many fluidic operations. They can be used for switching or adjusting flows between chemicals in a Y-junction geometry^3,4^, for mixing chemicals^5,6^, and for peristaltic pumping^7^. They are also widely used in droplet microfluidics^8^ to generate droplets from different chemicals^9^, to alter droplet size and frequency^10^, to split^11^, sort^12^, and to merge droplets ^13^.

Microfluidic valves have been utilised for powerful applications such as immunoassays^14^, drug screening^15^, binding assays^6^, nucleic acid purification^16^, and microchip electrophoresis^17^. While these are great examples of assays carried out on chips in a lab, it is essential to work towards rendering these capabilities portable for point-of-care applications.

Microfluidic valves are challenging to implement as they usually require moving parts embedded within micron-scale channels. Nevertheless, various types of valves have been reported in the literature, categorised here based on three actuation types: pneumatic, mechanical, and thermal.

Pneumatic valves are the most commonly used type of microfluidic valves as they are easy to fabricate and operate^18–20^. Pneumatic valves are integrated into chips made from polydimethylsiloxane (PDMS), a commonly used elastomer in microfluidics, via a 2-layer fabrication method involving a control layer for air and a fluidic layer separated by a thin membrane^21^. Control of pneumatic valves are either achieved by pressure regulators^11^ or solenoid valves^22^. While pressure regulators offer more control, allowing them to partially close^11^, they require expensive equipment. Solenoid valves, on the other hand, are low-cost (see Cost Analysis) and offer on/off functionality to control each valve individually. Multiple pneumatic valves could be operated with a single air compressor.

Mechanical valves incorporate a solid actuator to close the valve. They are often actuated by a piston which deforms a membrane, which in turn restricts flow in a microchannel^23,24^. One example is using a Braille pin device to actuate the valves^15,25^. In a recent study by Utharala et al.^26^, a Braille pin device was used to sort droplets into eight different outlets.

One of the most promising types of microfluidic valves for lab-on-a-chip applications is the thermally-actuated microvalve because they can be operated by a power supply and a microcontroller, making them strong candidates for point-of-care applications and large-scale integration^27^. Paraffin, owing to its volume expansion properties during phase change^28^, is commonly used as an expansion medium for thermally actuated valves^29–33^. Paraffin waxes have large actuation power capability^34^ and a low, tuneable melting point^35,36^. Fluorocarbon oil (FC-40) has also been used as the expansion medium^37^. Other types of thermally actuated valves involve chip-embedded thermoresponsive polymers such as hydrogel^38,39^ or ionogel^40^ which reversibly swell up to restrict flow based on their phase transition temperatures.

Presented here is a thermally actuated valve that uses olive oil as the expansion medium. The valve is demonstrated using a three-layer PDMS chip; in the first layer, the microfluidic structures are patterned, the second layer is a membrane layer and the final layer houses rigidity ports for inserting capillary tubes. The capillary tube is filled with olive oil and fitted with a resistance wire for heating. Finally, the other end of the capillary tube is sealed with epoxy glue for preventing leakage. Resistance wire heaters are used to heat the oil which expands to block flow in a microfluidic channel. In this paper, the operating principle of this valve is demonstrated and an application to adjust the chemical concentration of two chemicals is showcased using a Y-junction microfluidic chip. The valve is characterised in terms of its power requirement, temperature change and durability.

The presented valve offers significant advantages over previous works in the literature. Firstly, the valve is simple and fabricated with off-the-shelf components without the use of cleanroom facilities. Individual valve cost has been calculated to be less than $1 per actuator making this suitable for scaling out and for disposable devices. The maximum temperature change has been recorded as 11 °C whilst the fastest actuation time was measured to be 2 s. The durability of the valve has been shown for 65 h of cyclical operation with 585 actuations before failure. Multiple valves are controlled with minimal peripherals—a power supply unit and a microcontroller, therefore, the system is programmable and suitable for point-of-care applications. Herein, the mathematical framework for valve design is also discussed allowing prediction of future design parameters such volume of the expansion media, expected temperature increase and required power based on microchannel dimensions.

## System overview

**Fig. 1:**
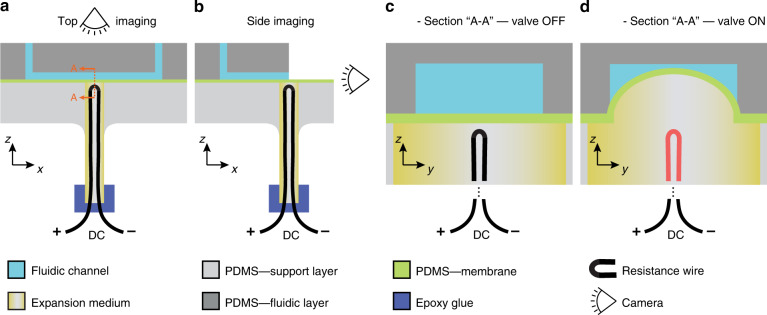
System overview. **a** Three-layer PDMS setup has the fluidic layer that houses the fluidic access ports and microchannels on the top. Middle layer houses the PDMS membrane that is ~200 μm thick; the bottom layer has a punched-hole with a glass capillary fitted. The capillary is filled with the expansion medium and a resistance wire is inserted for heating. The capillary is sealed with epoxy glue. Imaging is from the top as shown. **b** The side imaging option is shown. During side imaging, the fluidic layer is cut in the middle for visualising membrane deformation in real time. **c** Zoomed in A-A section view where the valve is in the OFF-state, no heating. **d** Zoomed in A-A section view where the valve is in the ON-state. Thermal expansion of the medium causes the membrane to block the flow in the fluidic layer

The three-layer PDMS structure is shown in Fig. 1a. In the top layer (dark grey), the microfluidic channels are patterned. The middle layer (green) is a PDMS membrane that is ~200 μm thick, the bottom layer (light grey) is a thick PDMS block which houses support ports for inserting capillary tubes. The capillary tube is filled with oil and a resistance wire is inserted for heating. The other end of the capillary is sealed with epoxy glue. Two types of imaging have been used, ‘top imaging’ and ‘side imaging’; camera positions are shown in Fig. 1a, b. When the valve is in OFF-state—no heating (Fig. 1c)—the microfluidic channels operate as normal. When DC power is applied to the resistance wire, the oil heats up, expands and restricts flow in the microfluidic channel (Fig. 1d).

**Fig. 2:**
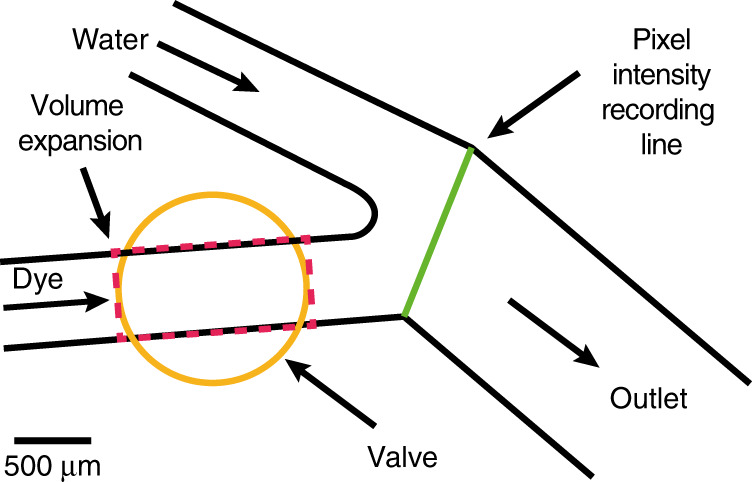
Microfluidic Y-junction chip for characterising the valve (entire chip design shown in Fig. S1). Water is pumped through one inlet while food dye is pumped through the other where the valve is integrated. The valve is located in the dye channel as shown with a circle. The dashed rectangle indicates the volume expansion area. A line is drawn in LabVIEW (shown as a green line) where the two flows meet; this is used to record pixel intensity data for further analysis

For proof-of-concept demonstration of the valve, top imaging (Fig. 1a) was used with a Y-junction microfluidic chip shown in Fig. 2 (entire microfluidic chip design shown in Fig. S1). The Y-junction microfluidic chip was supplied with water from one inlet while food dye was used in the other inlet where the valve is located. After the two flows meet, a pixel intensity recording line (see ‘Materials and methods’) is used to log data for analysis (Fig. 2). Peripheral configuration 1 has been used for this experiment, see ‘Materials and methods’ for details.

**Fig. 3:**
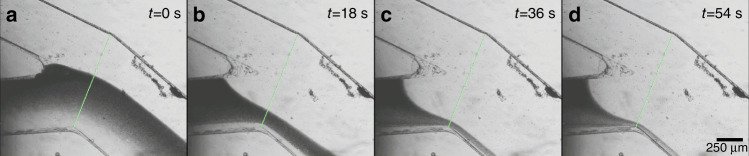
Time-lapse images showing the valve in action. **a** The valve is turned on at *t* = 0 s when the water–dye interface is roughly in the middle of the channel. **b**–**d** The oil in the valve heats up, expands and blocks the flow in the dye channel. The interface gradually recedes to a bare minimum

To demonstrate how the valve works, time-lapse images during valve operation are shown in Fig. 3. The heating is turned on at *t* = 0 s when the two interfaces meet stably in the middle of the bigger channel (Fig. 3a). As the oil heats up, it expands and applies pressure on the membrane to restrict flow coming from the dye inlet. Over time, the interface recedes to a bare minimum (Fig. 3b–d) as expected from a sieve valve^41^. Supplementary Video 1 shows the valve operation in real time.

## Results and discussion

### Theoretical power requirement

To characterise the microvalve presented in this paper, firstly, we focus on determining power requirements for turning on (heating) one valve. This is an important parameter for portable applications, especially those which employ a large number of valves. The power requirement is initially calculated theoretically and later compared with experimental results.

To theoretically calculate the power requirements for turning on one valve, we will start with the assumption that pressure in the heated oil section will be sufficient to overcome the pressure in the microfluidic channel. According to a mathematical model developed to estimate pressure increase due to thermal expansion of a trapped liquid^42,43^; the expected pressure rise is 10.88 bar per K (see Supplementary Information for details). This is an overestimate because the model assumes homogeneous material properties surrounding the blocked-in liquid, however, in this paper, one end of the capillary tube is sealed with epoxy glue and the other with PDMS membrane. Notwithstanding, 10.88 bar per K is more than enough to overcome a pressure of several hundred millibars, which is commonly used to drive microfluidic flows.

An estimate on the volume expansion required to block the flow in the microfluidic channel could be calculated as ΔV = 50 nL using the volume of a rectangular prism where the width and length are that of the microfluidic channel and the diameter of the valve (500 μm × 2000 μm) (shown as a dashed rectangle in Fig. 2) while the height is the height of the microfluidic channel (50 μm). The temperature change, ΔT, in the oil required to generate volume expansion equal to 50 nL can be calculated using the volume expansion formula^44^:

1 $${{\Delta }}T=\frac{{{\Delta }}V}{{V}_{0}\beta }$$

where *V*_0_ is the initial volume and *β* is the volume expansion coefficient of the oil. Next, we will use specific heat formula to find the required energy, *Q*, to change the temperature by Δ*T*:

2 $${{\Delta }}T=\frac{Q}{m{c}_{p}}$$

where *m* and *c*_*p*_ are the mass and the specific heat of the oil. To find power requirement, *W*, we use *Q* = *W*Δ*t* where Δ*t* is the time it takes to actuate the valve. We will also substitute the density formula into Eq. (2) given by *m* = *ρV*_0_ where *ρ* is the density of the oil. Substituting the above and equating Eqs. (1) and (2), we get:

3 $$W({{\Delta }}V,{{\Delta }}t)=\frac{\rho {c}_{p}}{\beta }\frac{{{\Delta }}V}{{{\Delta }}t}$$

It is interesting to note, assuming Δ*T* stays within an acceptable range (e.g. no boiling; this is revisited later in this section), the power requirement during heating is expressed as a function of fluid properties, the required volume change and the time it takes to turn on the valve (Eq. (3)). Relevant properties of the fluids used in this study are given in Table 1.

**Table 1:** Properties of the different actuation media that are relevant to this study

Property	Olive oil	Water	FC-40
Volume expansion coefficient, *β*, [K^−1^]	7.5 × 10^−4^ [Ref.^45^]	2.14 × 10^−4^	12 × 10^−4^
Specific Heat, *c*_*p*_, [J/(kg K)]	1970	4180	1100
Density, *ρ*, [kg/m^3^]	916	997	1850

**Fig. 4:**
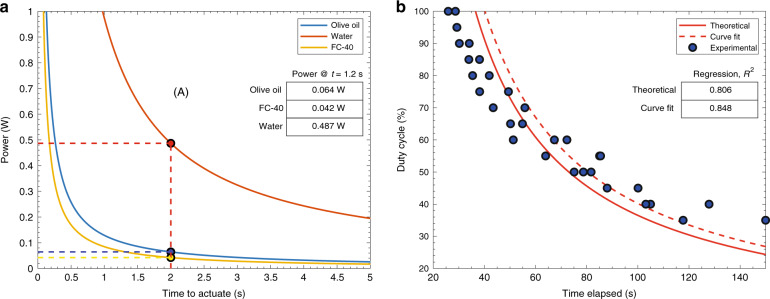
The relationship between applied power and time to actuate. **a** Power required to actuate one valve according to Eq. (3) using Olive Oil, FC-40 or water as the expansion medium. The inset table shows the required power values when time to actuate is 2 s. **b** Experimental results of time elapsed to reach a certain Δ*V* are shown as blue circles denoted ‘Experimental’. The expected duty cycle and time elapsed according to Eq. (3) is plotted as a solid line denoted ‘Theoretical’. Curve fit to the experimental data is shown as a dashed line denoted ‘Curve Fit’. The inset table shows *R*^2^ values for the curves

Using the required volume expansion and the properties of the oil used in this study as well as water for comparison, Fig. 4a shows the power requirement as a function of actuation time, Δ*t*. As can be seen, there is a significant trade-off between the required power and the actuation time. Also, were water to be used as the expansion liquid, it would have required much more energy due to the high specific heat of water.

### Experimental power requirement

To measure how long it takes to actuate the valve, a side imaging (Fig. 1b) experiment was carried out where the expansion and contraction of the valve along with the microchannel could be observed via a camera. Peripheral configuration 2 has been used for this experiment, see ‘Materials and methods’ for details. In this experiment, an equilibrium was sought where zero net heating energy into the expansion medium was targeted. Arduino microcontroller was programmed so that the heating was on for 1.2 s and off for 5.5 s. In these time-frames, it was observed that net energy into the expansion medium was close to zero. When the net energy was positive, the valve was observed to expand and the valve would stay closed during the cooling period. When the net energy was negative, the valve would cool down over time and would not block the microchannel any more. More importantly, during 1.2 s of heating and 5.5 s of cooling, it was observed that the expansion of the valve was enough to close the microchannel (see Supplementary Video 2).

To verify this, a constant-pressure, top imaging experiment has been carried out with peripheral configuration 3, see ‘Materials and methods’ for details. In this experiment, it was found that 1.2 s was not enough to fully close the valve, instead, 2 s of heating and 9 s of cooling was used to turn the valve on and off (see Supplementary Video 3). This difference is attributed to the additional thermal mass and pressure in the system due to the emulsion flowing inside the microchannel which was not the case for the side imaging experiment where the microchannel was open to air (Fig. 1b). Based on the top imaging experiment, the fastest time it takes to close or open the valve has been established as 2 s and 9 s, respectively.

A vertical dashed line has been shown on Fig. 4a corresponding to the closing time of 2 s. This line intersects the olive oil and water curves at 0.064 W and 0.487 W, respectively. From this, we infer that, theoretically, 0.064 W is required to fully close the valve. Power consumption during heating up was experimentally calculated by measuring the voltage across the heater, *V*_*h*_ and using previously measured resistance (room temperature) of the heated wire, *R*, according to ‘Joule heating’ formula given as $W={V}_{h}^{2}/R$. Experimental results show that 0.73 W is provided to the resistance wires when the heating is turned on with peripheral configuration 2 (see ‘Materials and methods’) at maximum power (100% duty cycle).

There is a significant discrepancy between the theoretical (0.064 W) and the experimental results (0.73 W). We believe this is explained by the losses in the system which are not accounted for in the theoretical calculations. The major losses in this study have been determined as heating of the wires and other components in the electrical circuit (see Fig. S2) along with heat lost to the environment. Finally, Δ*V* is expected to be larger than the calculated value of 50 nL (Eq. (3)) due to the elasticity of the PDMS. While the elasticity of the channels could be minimized by using another material such as glass, the heat lost to the environment is beneficial when it comes to re-opening of the demonstrated thermal valve.

To investigate Eq. (3) further, a side imaging (Fig. 1b) experiment was designed. Peripheral configuration 2 has been used for this experiment, see ‘Materials and methods’ for details. In this experiment, power was changed from 100% duty cycle to 35% duty cycle in increments of 5%. Below 35% duty cycle, the valve operation was simply too slow or the power was insufficient to overcome ambient cooling. At every power level, after 5-min cool-down, the time elapsed to reach a predetermined valve expansion was measured. This ensured that Δ*V* was kept constant across experiments. The expected power requirement and time elapsed according to Eq. (3) was plotted as a solid line (denoted ‘Theoretical’, Fig. 4b). The experimental data points are shown as blue circles denoted ‘Experimental’. At each power level, the measurement was repeated at least two times. It can be seen that the experimental results follow a similar trend to the expected curve. A regression analysis shows that the *R*^2^ value is 0.806 (Fig. 4b). Furthermore, the form of Eq. (3) with an adjustable constant of proportionality, *A*, (*W*(Δ*t*) = *A*/Δ*t*) was used for fitting a curve to the experimental data points; this is shown as a dashed line denoted ‘Curve Fit’. Regression analysis shows that the *R*^2^ value is improved and is 0.848 for the fitted curve (Fig. 4b).

### Chemical concentration

To showcase the tuning of a water/dye dilution mixture, a series of top imaging (Fig. 1a) experiments have been performed by measuring the interface settling location. Peripheral configuration 1 has been used for this experiment, see ‘Materials and methods’ for details. The Y-junction microfluidic chip (see Fig. 2 and Fig. S1) has been used. In this set of experiments, referring to Fig. S1, port 1 was supplied with water, port 2 was supplied with dye, port 5 was the outlet and ports 3–4 were closed. The flow rates on both inlets have been kept constant and the valve has been actuated at different power levels to allow the dye-water interface to settle in a final location. This way, the chemical concentration of the output is modified in a continuous flow microfluidic chip. Alternatively, peripheral configuration 2 (see ‘Materials and methods’) could be used to control the interface location real time using a feedback control loop. As an example, this could be used to generate droplets of varying chemical concentrations using a chip similar to the one described by Park et al. ^46^.

**Fig. 5:**
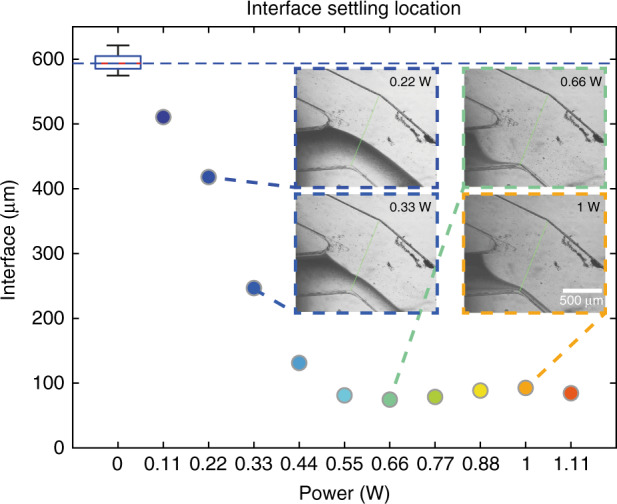
The effects of increasing power on the final interface settling location. In the absence of heating (0 W), the interface settles at around 600 μm; this is shown as a boxplot due to fluctuation in the precise location of the interface. As the applied power increases, the valve blocks the dye inlet and the interface recedes. The insets show experimental images of interface settling location from power levels of 0.22 W, 0.44 W, 0.66 W and 1 W. Scale bar is 500 μm

At each power level reported in this Fig. 5, steady-state interface location is reported. For some of the data points, the interface settling location is shown visually. Initially, there is no actuation and the system is at rest. At this state, the interface slightly fluctuates due to the stepper motor in the syringe pump. Therefore, the zeroth data point is shown as a box plot captured over 60 s at 12 fps in Fig. 5 (see ‘Experimentation and data analysis’ for further details). It can be seen that the interface settles slightly below 600 μm. The location of the interface along the intensity line is measured from bottom to top and the length of the intensity line is 1 mm; the initial settling is slightly biased for the dye channel (40–60% water–dye mixture). At 0.33 W input power (Fig. 5), the interface settles at around 250 *μ*m rendering the outlet a 75%–25% water–dye mixture. At higher power levels (>0.5 W), the dye flow is reduced to a bare minimum (<10%, Fig. 5 inset 0.66 W and 1 W); not fully closed as the channels are rectangular making this a sieve (leaky) valve ^41^.

To further investigate the leaky nature of the valve, a top imaging experiment has been carried out with peripheral configuration 3 where an emulsion flow—to visualize the flow—is driven by constant pressure (see ‘Materials and methods’). With a mid-level power applied to close the valve, it can be seen in Supplementary Video 4 that the flow is leaky around the edges of the microchannels until the valve fully blocks and stops flow successfully (*t* = 1:13 s, Supplementary Video 4). It’s been observed that flow remained leaky during the experiments with syringe pumps (Fig. 3(D), therefore, the successful closure is attributed to the constant-pressure driven flow. The leakiness is caused by the mismatch between the rectangular microchannel cross-section and hemispherical membrane (Fig. 1d). In the literature, it’s been shown that rounded channel structures could be used for fabricating fully closing valves^47^; rounded microchannels could be obtained via wet etching glass microfluidic chips or reflowing photoresist^48^. Lee et al.^47^ have used sieve (leaky) valves and fully closing valves together in a microfluidic device where the sieve valves have been used to trap bigger particles, anion exchange beads, while still allowing flow to filter through thus creating an anion exchange column.

### Multiple valves

Supplementary Video 5 shows two valves operating in succession. Side imaging (Fig. 1b) and peripheral configuration 2 (see ‘Materials and methods’) was used to carry out these experiments with the exception that the fluidic layer was not installed. There were three valves during this experiment, however, only two of them could be visualised at the same time. The power was supplied was from the 12 V output of a computer power supply (GPT500S, Channel Well Technology Co., Ltd.). Metal Oxide Semiconductor Field-Effect Transistors (MOSFETs) were used as switches to turn the valves on or off. MOSFETs are controlled by a voltage signal applied at the gate terminal; this was provided by digital pins with pulse-width modulation (PWM) capability on an Arduino Uno microcontroller board. Arduino Uno offers 6 PWM pins, however, Arduino Mega offers 15. The power supply is capable of providing 456 W on the 12 V rail which can operate more than 100 valves. This shows that the scaling out with the proposed valves is feasible on a technical and cost point-of-view.

### Durability

Supplementary Video 6 shows a side imaging (Fig. 1b) experiment carried out for testing the durability of a valve. Peripheral configuration 2 (see ‘Materials and methods’) was used for this experiment. The valve was heated for 200 s and cooled for 200 s. An image was captured every 200 s so that consecutive images of the valve being on and off could be seen. Supplementary Video 6 is constructed as a time-lapse video (30 fps) from the 1295 images captured, the experiment approximately ran for 3 days. The valve failed at image 1170; after 585 actuations in 65 h of operation (see Supplementary Videos for further details). For a disposable chip aimed at point-of-care assays, it is believed that this durability will be sufficient. The main mode of failure has been observed as leaking due to local heat damage to the epoxy glue (Fig. 1a). This could potentially be alleviated by using a high-temperature resistant epoxy to improve the durability further, however, it should be noted that epoxy glue represents 40% of the total cost of one valve, therefore, this might have an impact on the overall cost of the proposed valve.

### Temperature change

**Fig. 6:**
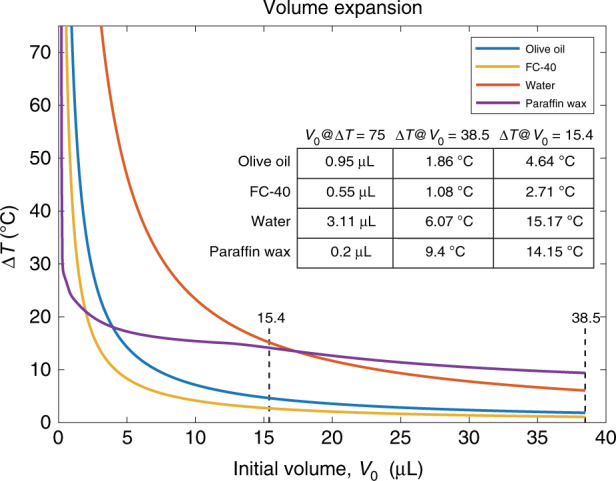
Volume expansion plot show the required temperature change as a function of initial volume for commonly used expansion media—olive oil, FC-40, water and paraffin wax. The inset table shows the initial volume when the temperature change is 75 °C (left) and the temperature change when the initial volume is at the values with which the experiments have been carried out

Next, we will investigate the temperature change required by the presented system. Recalling how the required temperature change, Δ*T*, was cancelled out to obtain Eq. (3), it is necessary to ensure that Δ*T* is an acceptable value (e.g. no boiling). To verify this, we use Eq. (1) to plot temperature change as a function of the initial volume, *V*_0_ (Fig. 6). Here, we assume 25 °C room temperature and limit Δ*T* at a maximum of 75 °C as this corresponds to the boiling point of water. It is expected that boiling in the expansion medium will be catastrophic for thermally actuated valves. Herein, we only consider the following media: olive oil, FC-40, water and paraffin wax. Fig. 6 shows Δ*T* up to 75 °C for these substances. The inset shows a table for all the substances noting the *V*_0_ value when Δ*T* = 75 °C as well as the Δ*T* value for *V*_0_ = 15.4 μL and 38.5 μL. These are the expansion media volumes with which the experiments have been carried out (Table 2). The expected temperature change for the 15.4 μL and 38.5 μL valves are 4.64 °C and 1.86 °C, respectively. In this work, minimal temperature increase is targeted for the valve to safely work with sensitive biological samples. Using the mathematical framework provided in this paper, thermally actuated valves with different characteristics could be designed. For example, if the valve will be deployed in the field in a hot environment, it could be designed so that the required temperature change (Δ*T*) is high to ensure it will not spontaneously close. Conversely, low temperature change (Δ*T*) could be preferred for operating in cold environments.

**Table 2:** Valve size variations

Property	Long valve	Short valve
Capillary length (mm)	25	10
Capillary outer diameter (OD) (mm)	2	2
Capillary inner diameter (ID) (mm)	1.4	1.4
Expansion media volume (μL)	38.5	15.4
Length of resistance wire (mm)	450	100
Length of resistance wire in oil (mm)	50	20

Measuring these values experimentally for comparison proved to be challenging with the current setup, however, an experiment was carried out to measure the maximum temperatures that could be reached with the current setup. For this experiment, a thermocouple was placed right above the valve, where the microfluidic channel would be. Side imaging (Fig. 1b) and peripheral configuration 2 (see ‘Materials and methods’) was used for this experiment. With a duty cycle of 100%, the valve was kept on for 40 s and the maximum temperature increase was measured as 11 °C by the thermocouple connected to a multimeter (1 °C precision, RS PRO RS14, RS Components Ltd., UK).

While paraffin wax, amongst the other media considered in this paper, has worse performance at higher initial volumes as shown by the higher temperature changes required in the inset table in Fig. 6, it outperforms the other expansion media when operated near its melting point. This should be considered when designing thermally actuated valves using paraffin as the expansion medium. The melting point of paraffin could be modified by changing the hydrocarbon composition^35^; it is around 40 °C (Δ*T* = 15 °C) for the composition reported by Ogden et al.^49^. Paraffin is, therefore, better suited to applications with low initial volume requirement.

For example, the thermally actuated valve using paraffin as the expansion medium reported by Carlen et al.^29,34^ features a volume (*V*_0_) of 1.26 nL. When heated up to ~90 °C, it is shown to deflect 3 μm. In the work reported by Pitchaimani et al.^37^, the expansion medium is fluorocarbon oil (FC-40) with a volume of 0.79 μL; the required Δ*V* is reported as 8.2 nL while the minimum temperature change was 27 °C. Actuation times ranged from 7 to 80 s when applied power was between 50 and 80 mW. For comparison, with olive oil as the expanding medium, this work features a Δ*V* of 50 nL, expected temperature change of 5 °C and a measured maximum temperature increase of 11 °C whilst the fastest actuation time was measured to be 2 s.

## Conclusion

This paper reports a low-cost, programmable, thermally-actuated microfluidic valve constructed using off-the-shelf components without requiring cleanroom facilities. The cost of a single actuator has been calculated to be less than $1 per actuator. The valve operation is demonstrated using a 3-layer polydimethylsiloxane (PDMS) chip with a fluidic layer, a membrane layer and a support layer for capillary tube inserts. The capillary tubes are filled with oil and fitted with a resistance wire for heating. As the oil heats up, it expands and deforms the membrane that blocks the microfluidic channel. An Arduino controlling MOSFETs was used with a computer power supply to operate multiple valves reducing the overall footprint of this system substantially. This is presented as an important step during the transition from chip-in-a-lab devices to portable, point-of-care devices.

This paper demonstrates how the microfluidic valve works and showcases how the final chemical concentration of a mixture can be adjusted in a continuous flow microfluidic chip. The valve is characterised by investigating the theoretical and experimental power requirements to operate one valve. While theory estimates 0.107 W, experimental results suggest 0.73 W is required to fully close the valve. The discrepancy is attributed to the losses neglected in the system, such as the energy dissipation due to thermal loss as well as the elasticity of the PDMS microfluidic chip.

The durability of the valve has been tested for 65 h of cyclical operation with 585 actuations. For the safety of biological materials, the expected temperature change in the expansion media is calculated to be less than 5 °C. At full power, the fastest closing (heating) and opening (cooling) time for the valve has been reported as 2 s and 9 s, respectively. Experimental results from operating the valve at full power for 40 s indicate that the temperature increase is 11 °C.

This paper presents an important step towards designing a microfluidic valve that is programmable and has minimal requirements for other peripheral equipment, which is a boon for portability. This is especially important for microfluidic detection platforms that are to be employed in remote locations with little or no access to advanced lab facilities. Microfluidic valves are amongst the most important building blocks of microfluidic devices, therefore, they should be at the forefront of the transition from chip-in-a-lab to lab-on-a-chip devices.

## Materials and methods

### Fabrication

The microfluidic device consisted of 3 layers of polydimethylsiloxane (PDMS) (Fig. 1a). The top layer houses the microfluidic channels. Microfluidic channel designs were transferred onto a silicon wafer using a chrome photomask and UV photolithography. Photoresist (SU-8 50, MicroChem Corp., USA) was spun onto a 4-inch silicon wafer at 2000 rpm. It was then pre-baked on a hot plate at 65, 95, and 65 °C for 2, 6, and 2 min, respectively. The resist was exposed to UV light (250 mJ/cm^2^) through the photomask with the channel structures. Post-exposure bake was carried out at the same temperatures for 1, 5, and 1 min. The resist was developed in propylene glycol methyl ether acetate for 2 min in an ultrasound bath. PDMS was prepared (10:1), cast onto the moulds and cured overnight at 60 °C. The middle membrane layer (Fig. 1a) was prepared by spin coating 1 gram of uncured PDMS deposited onto a 90 mm petri dish and curing it. The bottom support layer (Fig. 1a) was simply blank PDMS (5 mm thickness) cured in a petri dish. Both the top and the bottom PDMS layers were ported using biopsy punches of 1 and 2 mm diameters, respectively.

PDMS was bonded onto PDMS by 30 s of oxygen plasma activation (180 mTorr) using PE-25 (Plasma Etch, Inc.). Firstly the top layer was bonded onto the membrane layer, later the membrane layer was bonded with the bottom layer to create a 3 layered PDMS device. Channel height was measured via cross-section microscopy as ≈ 50 μm.

A capillary (various sizes, see Table 2) was inserted into the punched hole in the bottom layer and sealed around with uncured PDMS. The capillary was filled with olive oil (La Espanola, Sainsbury’s Ltd, UK) and trapped air was removed with a vacuum desiccator. The resistance wire (0.132 mm 39 SWG Enamelled Nickel Chrome Wire, Eurowire Ltd.) was cut into various lengths (see Table 2) and inserted into oil after folding in half. Only a certain length of the resistance wire was inside the oil (see Table 2, the reported power values took that into account. The open end of the capillary was sealed with a 2-part epoxy (F112, Thorlabs) that fully cured at room temperature in 24 h.

### Peripheral configuration 1

Peripheral configuration 1 was used for the reported results except for the results shown in Fig. 4. The microfluidic chip was operated via 2 syringe pumps (AL300-22, World Precision Instruments Ltd.). Readers should be aware that this could lead to a build-up of pressure when the valve is turned on and blocking the microfluidic channel; therefore for certain applications, constant-pressure pumps may be better suited to this type of valve. 1 mL BD Plastipak syringes were filled with de-ionized water and undiluted black food dye (black food colouring, Sainsbury’s Ltd, UK). Syringes were then loaded onto the syringe pumps and connected to microfluidic chips via polyethene tubing (~50 cm). The water flow rate was set to 20 μL/h and the dye flow rate was set to 160 μL/h during experiments. The flow rates were controlled to produce a stable water–dye interface that was approximately in the middle of the channel. Long valves were used (Table 2) to actuate. The microfluidic channels were imaged from the top (see Fig. 1a). A microscope built from a 4× microscope objective (Olympus PLN4X) and tube lens (Olympus U-TLU) was used. Images were captured using a monochrome camera (UI-3080CP Rev. 2, Imaging Development Systems GmbH) while transmission illumination was provided using an LED torch. Resistance wires were heated up with a power supply (PL303-P, Aim-TTi), the voltage and current data were acquired through a data cable using a custom LabVIEW interface.

### Peripheral configuration 2

Peripheral configuration 2 was used for the reported results shown in Fig. 4 and Supplementary Videos 2, 5 and 6. Short valves were used (Table 2). The valves were imaged from the side with a section-cut microfluidic channel (see Fig. 1b). A stereo microscope equipped with a monochrome camera (UI-3080CP Rev. 2, Imaging Development Systems GmbH) was used to acquire images. Transmission illumination was provided using an LED ring. The actuation of the valves was controlled by MOSFETs switched by a microcontroller (Arduino Uno). The circuit diagram is shown in Fig. S2. Power was supplied by the 12 V output on a computer power supply unit (GPT500S, Channel Well Technology Co., Ltd.).

### Peripheral configuration 3

Peripheral configuration 3 was used for Supplementary Videos 3 and 4. Short valves were used (Table 2). The microchannels were imaged from the top (see Fig. 1a). Constant pressure pumping was used instead of syringe pumps. A 5 mL syringe was filled with a vortexed water-in-oil emulsion (1:1(v:v)) of an engineered fluid (3M^TM^ Novec^TM^ 7500) stabilised by 1% surfactant (FluoSurf, Emulseo) and DI-water. The syringe was secured at a specific height with the plunger pulled out. The distance from the top of the liquid to the open end of the outlet tube was set to 500 mm corresponding to ~50 mbar pressure differential. A stereo microscope equipped with a colour camera (BFS-U3-32S4C-C, FLIR Systems, Inc.) was used to acquire images. Transmission illumination was provided using an LED ring. The actuation of the valves was controlled by MOSFETs switched by a microcontroller (Arduino Uno). The circuit diagram is shown in Fig. S2. Power was supplied by the 12 V output on a computer power supply unit (GPT500S, Channel Well Technology Co., Ltd.).

### Experimentation and data analysis

Images from the camera were acquired by custom LabVIEW software during experimentation. A line was drawn on the live image across the microfluidic channel as shown in Figs. 2, 3, and 5. Using LabVIEW’s ‘IMAQ LineProfile VI’, the light intensity of each pixel along the line was obtained. This data, along with timestamps, was saved as text files and imported into MATLAB for analysis. The line intensity profile obtained is similar to a sigmoid curve. The location of the interface was obtained by selecting a light intensity value that was 75% of the difference between the dark and the light regions. Data analysis for experiments reported in Figs. 3 and 5 were conducted in this way.

During experiments for Fig. 5, when the valve was not actuated, the location of the interface fluctuated, therefore, the first data point is given as a boxplot after the interface was observed for 60 s at 12 fps. When the valve was actuated, the interface location decreased over time. The slope of the interface location was smoothed using a moving average of 100 data points. The interface was considered to have settled when the smoothed slope reached −1. The experiments were carried out once for different power levels.

During experiments for Fig. 4b and Supplementary Video 2, where ‘Peripheral configuration 2’ was used, Microsoft PowerPoint’s Screen Recording (10 fps) tool was used. On the recorded screen was the image from the stereo microscope acquired by LabVIEW along with a timestamp as well as 2 channels from an oscilloscope (PicoScope 2204A, Pico Technology) and Arduino programming interface (see Supplementary Video 2).

There are two values reported in Fig. 4b, duty cycle and time elapsed. Duty cycle is input by the user in the Arduino code where a maximum of 255 corresponds to 100% duty cycle. Duty cycles from 35% to 100% with increments of 5% were used for these experiments (Arduino input rounded to the nearest integer). At every duty cycle level, the time elapsed was calculated by subtracting the endpoint timestamp from the onset timestamp. The onset timestamp for actuation was captured in a frame-by-frame analysis of the screen recording. At the onset, the signal from Oscilloscope Location 3 (see Fig. S2) changed from a flat 0 V signal to a pulse width modulated signal between 0 V and 4 V. The endpoint was determined when the membrane bulge reached a certain point that was predetermined and preserved across experiments; in other words, Δ*V* (Eq. (3)) was kept constant.

Paraffin wax data reported in Fig. 6 was obtained from Fig. 3 (0 MPa) in Ogden et al.’s review paper^49^ using WebPlotDigitizer. The specific volume data were interpolated using MATLAB’s 1-D interpolation with shape-preserving piecewise cubic interpolation option and linearly extrapolated down to room temperature (25 °C).

### Supplementary Videos

Supplementary Video 1 shows an experiment (peripheral configuration 1) with the Y-junction microfluidic chip (Fig. S1) during a water and dye co-flow. The dye flow is restricted when the valve is turned on.

Supplementary Video 2 shows an experiment (peripheral configuration 2) carried out to measure the maximum frequency at which the valve could be turned on and off. This video is captured using Microsoft PowerPoint’s Screen Recording (10 fps) tool. On the recorded screen was the image from the stereo microscope acquired by LabVIEW along with a timestamp as well as 2 channels (A&B) from the oscilloscope and Arduino programming interface. Channel A (blue) was connected to Oscilloscope Location 1 (Fig. S2) measuring the steady 12 V output from the power supply. Channel B (red) was connected to Oscilloscope Location 3 (Fig. S2) for getting a reference gate signal that was identical to the one sent to the MOSFET controlling the valve that the microscope was imaging.

Supplementary Video 3 shows an experiment (peripheral configuration 3) carried out to measure the maximum frequency at which the valve could be turned on and off. This video is captured using Microsoft PowerPoint’s Screen Recording (10 fps) tool. On the recorded screen was the image from the stereo microscope acquired by LabVIEW along with Arduino programming interface. The valve was programmed to stay on (heat) at max power for 2 s and off (cool) for 9 s repeatedly. The channel was filled with a water-in-oil emulsion for tracing flow. The flow was driven via a constant pressure differential between the inlet and the outlet (50 mbar).

Supplementary Video 4 shows an experiment (peripheral configuration 3) carried out to investigate the leaky nature of the valve. This video is captured using Microsoft PowerPoint’s Screen Recording (10 fps) tool. On the recorded screen was the image from the stereo microscope acquired by LabVIEW along with Arduino programming interface. The valve was programmed to stay on (heat) (at 60% duty cycle) for 45 s and off (cool) for 10 s. The channel was filled with a water-in-oil emulsion for tracing flow. The flow was driven via a constant pressure differential between the inlet and the outlet (50 mbar).

Supplementary Video 5 shows two valves operating in succession. Peripheral configuration 2 (Fig. 1b) was used to carry out these experiments with the exception that the fluidic layer was not installed for visualising the valves from the side.

Supplementary Video 6 shows an experiment carried out for testing the durability of a valve. The valve was turned on for 200 s and off for 200 s. An image was captured every 200 s so that consecutive images of the valve being on and off could be seen. Peripheral configuration 2 (Fig. 1b) was used for this experiment. Supplementary Video 6 is a time-lapse video (30 fps) composed of 1295 images, the experiment approximately ran for 3 days. The valve failed at image 1170 after 65 h of being turned on and off. The on and off signal was programmed in Arduino, however, images were captured with a custom LabVIEW program. The timings of the two did not match precisely, this lead to a shift in the captured images. This is why the valve appears to be in slightly different positions in the video.

### Cost analysis

#### Peripheral equipment

In ‘Peripheral Configuration 2’ (see ‘Materials and methods’), we have used a computer power supply unit (PSU) (GPT500S, Channel Well Technology Co., Ltd.) to actuate the presented valves. PSUs are widely available and are relatively cheap ($56). The 12 V output on the PSU was used to power the Arduino Uno ($25, up to 6 PWM + 8 ON/OFF valves). The microcontroller and the PSU could be upgraded to handle many more valves if required.

#### Valves

Solenoid valve prices vary significantly, they can be found for as little as $7.5. The one used in Lee et al.^50^, and Stephenson et al.^51^, is listed for ~$40. For thermally actuated valves operated by microfabricated heaters^34^, it is hard to estimate the cost for microfabricated heaters as they can be batch-processed, however, access to cleanroom facilities, usage of specialised equipment and materials make it arduous. For Braille pin devices, the device with 64 pins and its controller is costed for $947^26^. For the actuator used in this study (Long Valve, Table 2), a capillary tube costs 4.5 cents, 40 μL of olive oil costs hundredth of a cent, the resistance wire costs 3.5 cents and the epoxy costs 40 cents bringing the total to less than 50 cents per actuator. For controlling actuators, a MOSFET ($0.4 per valve, IRF510, Vishay Intertechnology, Inc.) was used along with a flyback diode ($0.04 per valve, 1N4001, Vishay Intertechnology, Inc.) to prevent inductive discharge. A circuit diagram is available in Fig. S2. Cost of connection cables has not been included. Factoring in the MOSFET and the diode per valve mentioned above, the total cost comes to less than $1 per actuator.

#### Microfluidic chip

PDMS was used to manufacture the microfluidic chips used in this study. PDMS has significant disadvantages such as contaminating equipment whilst uncured, therefore, it is rarely used for commercial-grade products. Nonetheless, for completeness, we estimate that our proof-of-concept chips cost ~$2 per chip excluding the initial fabrication of the mould. For cheaper mass manufacturing, COC or PMMA could be used; for better chemical resistance, whole-Teflon chips^19^ or glass microfluidic chips^52^ with Viton^®^ membranes can be used ^53^.

## Supplementary information

Supplementary Information

Supplementary Video 1

Supplementary Video 2

Supplementary Video 3

Supplementary Video 4

Supplementary Video 5

Supplementary Video 6

## Data Availability

All connected data to this manuscript is available from the authors upon reasonable request.
