# Supplementary material for: Thermally-actuated microfluidic membrane valve for point-of-care applications
Source: Microsyst Nanoeng. 2021 Jun 15;7:48. doi: 10.1038/s41378-021-00260-3 (PMC8433387; doi:10.1038/s41378-021-00260-3)
Supplement: Supplementary file 1 — Supplementary Information [file 41378_2021_260_MOESM1_ESM.pdf]

Supplementary Information
Thermally-actuated microfluidic membrane valve for point-of-care applications

Muhsincan Sesen[1] and Christopher J. Rowlands[1,*]

[1]Imperial College London, Department of Bioengineering, London, SW7 2AZ, United Kingdom
[*]Corresponding Author: Christopher J. Rowlands, Email: c.rowlands@imperial.ac.uk

1 Pressure Increase due to Thermal Expansion

In this section, we calculate the estimated pressure increase in the valve due to thermal expansion. The simultaneous heating of the pipe and the blocked-in liquid leads to a pressure build-up that could be catastrophic for macroscale piping systems such as oil and natural gas lines that are exposed to the sun. Hence, accurate mathematical models have been developed. The theoretical pressure increase due to thermal expansion per 1 K (dP/dT) temperature rise is given by the following equation:[1,2]

$$\frac{dP}{dT} = \frac{(\beta_L - 3\alpha_S)E_S}{E_S \kappa_L + (\frac{D}{\delta})(1.25 - \nu_S)} = 10.88 \text{ bar K}^{-1} \tag{S1}$$

This equation assumes a cylindrical vessel filled with a compressible liquid. This model assumes the enclosed vessel is made of homogeneous material, however, in the case of our experiments, one end of the capillary tube is sealed by an epoxy glue and the other end is sealed by a PDMS membrane. It is expected that the pressure rise in the expansion medium is less than 10.88 bar/K, however, still sufficient enough to overcome pressures of several hundred millibars, which is commonly used to drive microfluidic flows. Subscripts L and S denote the liquid and solid piping wall, in this case, olive oil and glass capillary tube. For liquid properties, 25°C and 1 atm are used, see Table S1 for the values used.

2 Microfluidic Chip Design

The full microfluidic chip design that is used in the experiments is shown in Fig. S1. There are 5 ports labelled with numbers. In the experiments that use this microfluidic chip, port 1 was supplied with water, port 2 with black food dye and port 5 was used as the outlet. Ports 3 and 4 were blocked.

3 Arduino Control Circuit

The electronic circuit diagram for controlling multiple valves is shown in Fig. S2. For simplicity, the diagram shows three heaters; every heater has a Metal Oxide Silicon Field Effect Transistor (MOSFET) to control on and off function as well as a flyback diode to prevent inductive voltage discharge during pulse width modulation (PWM). PWM is provided by PWM enabled pins on the Arduino microcontroller board. The 12V rails of a computer power supply unit (PSU) has been used to provide power to the heaters. The oscilloscope has been installed to three separate locations for measurements as shown in

Table S1. Material properties

Property	Olive Oil	Glass
Thermal Expansivity, β_L, [K^{-1}]	7.5×10^{-4} [Ref.[3]]	-
Linear Thermal Expansion Coefficient, α_S, [K^{-1}]	-	5.9×10^{-6}
Young's Modulus, E_S, [Pa]	-	70×10^{9}
Isothermal Compressibility, κ_L, [Pa^{-1}]	5.75×10^{-10} [Ref.[3]]	-
Diameter of the capillary tube, D, [m]	-	2×10^{-3}
Wall thickness of the capillary tube, δ, [m]	-	300×10^{-6}
Poisson's Ratio, ν_S	-	0.22

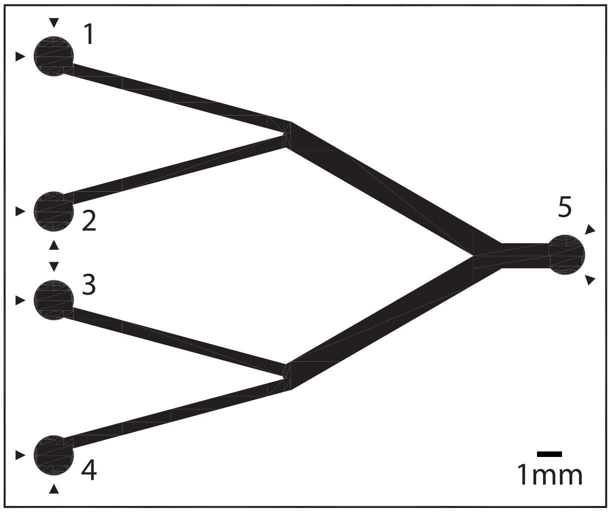

Figure S1. The full microfluidic chip design used in the experiment.

the diagram. Location 1 is used to monitor the 12V output from the PSU, location 2 is used for measuring the voltage drop across the heater and location 3 is used as a reference signal for monitoring the PWM output. While programming the Arduino microcontroller, the code for any heater pins (9, 10, 11) can be duplicated for pin 6 for monitoring the output behaviour of the PWM pins.

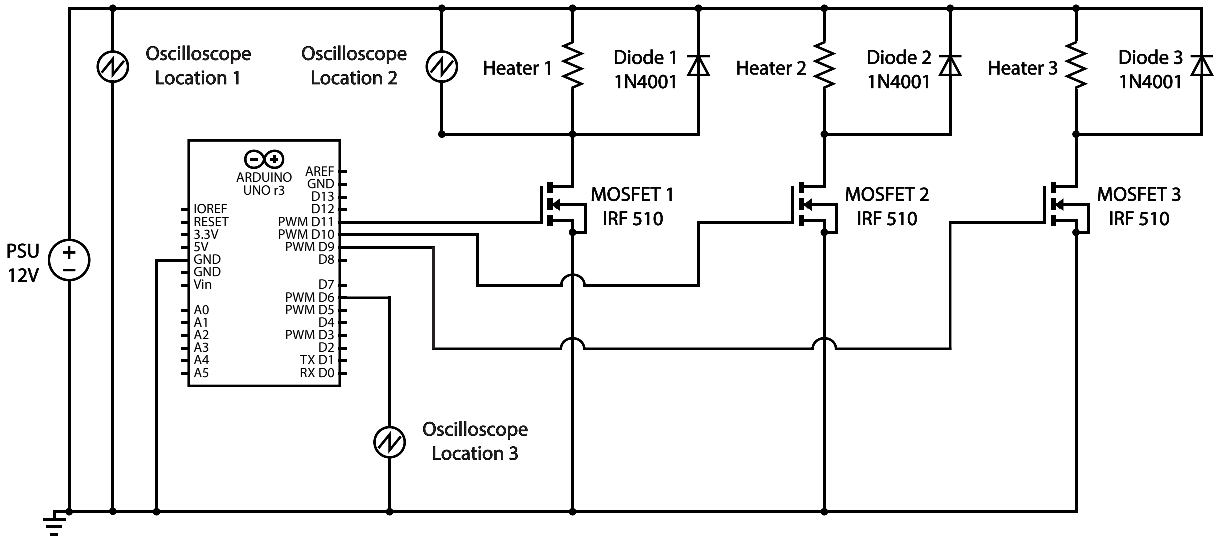

Figure S2. The electronic circuit diagram for controlling multiple valves. Flyback diodes prevent inductive voltage discharge during pulse width modulation.

References

1. Karcher, G. C. Pressure changes in liquid filled vessels or piping due to temperature changes. *ExxonMobil Research and Engineering Mechanical Newsletter* **EE.84E.76** (1976).
2. Center for Chemical Process Safety (CCPS), C. Guidelines for pressure relief and effluent handling systems (American Institute of Chemical Engineers, 1998).
3. Guignon, B., Aparicio, C. & Sanz, P. D. Volumetric properties of sunflower and olive oils at temperatures between 15 and 55 C under pressures up to 350 MPa. *High Pressure Research* **29**, 38–45 (2009).
